# Association of Childhood Chronic Physical Aggression with a DNA Methylation Signature in Adult Human T Cells

**DOI:** 10.1371/journal.pone.0089839

**Published:** 2014-04-01

**Authors:** Nadine Provençal, Matthew J. Suderman, Claire Guillemin, Frank Vitaro, Sylvana M. Côté, Michael Hallett, Richard E. Tremblay, Moshe Szyf

**Affiliations:** 1 Department of Pharmacology & Therapeutics, McGill University, Montreal, Quebec, Canada; 2 Research Unit on Children's Psycho-Social Maladjustment and Sainte-Justine Hospital Research Center, University of Montreal, Montreal, Canada; 3 Sackler Program for Epigenetics and Psychobiology, McGill University, Montreal, Quebec, Canada; 4 Department of Psychology and Pediatrics, University of Montreal, Montreal, Quebec, Canada; 5 School of Public Health, Physiotherapy and Population Sciences, University College Dublin, Dublin, Ireland; 6 McGill Centre for Bioinformatics, McGill University, Montreal, Quebec, Canada; 7 School of Psycho-Education, University of Montreal, Montréal, Quebec, Canada; 8 School of Social and Preventive Medicine, University of Montreal, Montréal, Quebec, Canada; The Nathan Kline Institute, United States of America

## Abstract

**Background:**

Chronic physical aggression (CPA) is characterized by frequent use of physical aggression from early childhood to adolescence. Observed in approximately 5% of males, CPA is associated with early childhood adverse environments and long-term negative consequences. Alterations in DNA methylation, a covalent modification of DNA that regulates genome function, have been associated with early childhood adversity.

**Aims:**

To test the hypothesis that a trajectory of chronic physical aggression during childhood is associated with a distinct DNA methylation profile during adulthood.

**Methods:**

We analyzed genome-wide promoter DNA methylation profiles of T cells from two groups of adult males assessed annually for frequency of physical aggression between 6 and 15 years of age: a group with CPA and a control group. Methylation profiles covering the promoter regions of 20 000 genes and 400 microRNAs were generated using MeDIP followed by hybridization to microarrays.

**Results:**

In total, 448 distinct gene promoters were differentially methylated in CPA. Functionally, many of these genes have previously been shown to play a role in aggression and were enriched in biological pathways affected by behavior. Their locations in the genome tended to form clusters spanning millions of bases in the genome.

**Conclusions:**

This study provides evidence of clustered and genome-wide variation in promoter DNA methylation in young adults that associates with a history of chronic physical aggression from 6 to 15 years of age. However, longitudinal studies of methylation during early childhood will be necessary to determine if and how this methylation variation in T cells DNA plays a role in early development of chronic physical aggression.

## Introduction

Longitudinal studies with birth cohorts have shown that children start to use physical aggression by the end of the first year after birth and frequency peaks between 2–4 years of age [1]–[4]. Longitudinal studies with school age children have found that the frequency of physical aggression decreases for the majority of children between 5 and 15 years of age [5]. However, a minority of children (4–7%) maintain a high frequency of physical aggression from childhood to adolescence [4]–[6]. Males on this chronic physical aggression (CPA) trajectory tend to grow-up in adverse family environments [4], [7]–[9], have lower cognitive abilities [10], tend to be rejected by their peers from early childhood onwards [11] and have numerous physical, mental and social problems such as accidents, hyperactivity, school failure, substance abuse and unemployment [4], [5], [10], [12]–[14].

Twin studies suggest that frequency of childhood physical aggression has a substantial inherited component [15]–[19]. At the molecular level, several polymorphisms were found to be associated with aggressive behavior in humans and animals [20]. Moreover, genetic and environmental factors have been shown to interact in the expression of impulsive aggression in monkeys [15] and violence in humans [21], [22]. However, very little work has been done to identify the mechanisms that might be responsible for these links. We hypothesize that DNA methylation is one such mechanism [4], [23].

It is now well-established that DNA sequence is complemented by epigenetic information including DNA methylation and histone modifications to program gene expression [24]. There is a growing body of evidence suggesting that in addition to the innate endogenous processes sculpting the DNA methylation pattern during gestation, the DNA methylation pattern is responsive to external environmental exposures including the social environment during both intra-uterine development and after birth [25] in animals [26]–[34] and in humans [35]–[37]. For example, early nurturing experiences influence epigenetic programming of the glucocorticoid receptor gene promoter in the hippocampus of rats [31] and humans [37].

DNA methylation patterns are tissue specific and it is therefore anticipated that changes relevant to behavior will only be detected in the brain [38]. Importantly however, DNA methylation alterations associated with social exposures are not restricted to the brain. Previous studies have shown associations between white blood cell (WBC) DNA methylation and various environmental exposures [39]–[42]. Borghol et al., have recently described association between early life socioeconomic position and DNA methylation signatures in adult WBC DNA [43]. Methylation of the *ADCYAP1R1* gene in peripheral blood DNA was found to be associated with post-traumatic stress disorder (PTSD) [44] and methylation of *FKBP5* in lymphocytes was associated with both genetic risk for PTSD and early life adversity [45]. Importantly, we have recently shown that differential DNA methylation of the serotonin transporter gene promoter (SLC6A4) in T cells and monocytes is associated with *in vivo* measures of human brain serotonin synthesis and childhood limited physical aggression in men [46]. Moreover, we have shown that young adult males on a chronic physical aggressive (CPA) trajectory between age 6 and 15 years had differential DNA methylated regions located in the genomic loci of cytokines and related transcription factors in T cells and monocytes, compared to males with the same background who did not follow such a high aggression trajectory (control group) [47], [48].

In the study presented here, we tested the hypothesis that a trajectory of chronic physical aggression during childhood would be associated with a distinct DNA methylation profile during adulthood. We compared blood CD3+ T cells genome-wide promoter methylation profiles of two groups: adult males who had been shown to be on a CPA trajectory between 6 and 15 years of age and males with the same background who followed a normal physical aggression trajectory (control group) [9], [12].

## Materials and Methods

### Participants

The subjects were recruited from participants in two longitudinal studies of child development [9], [49]. We recruited two groups of Caucasian men who were born in families with a low socioeconomic status and were living at the time of the present study within 200 km from our laboratory. The first group had a history of high physical aggression from age 6 to 15 years (chronic physical aggression group, CPA). The second group was recruited from the same longitudinal studies but included only those who did not have a history of high physical aggression from age 6 to 15 (Control group). A total of 65 eligible subjects agreed to participate (8 CPA and 57 controls). All of the 8 CPA subjects were included in the study and for budgetary reasons we reduced the control group to 12. In addition to physical aggression, other behavioral problems, such as hyperactivity, were also rated from age 6 to 15 and violence at 21 years of age. Characteristics of the 2 groups are presented in Table 1 (Additional information on the characteristics of the group can be found in File S1).

**Table 1:** Characteristics of the chronic physical aggression (CPA) group and control group.

Variables	Control		CPA		Group
	Mean ± SD or % (n)				
Age at blood drawn	25.4±2.71	*(12)*	25.8±2.87	*(8)*	*t*(18) = −0.26, *P* = 0.80
Familial adversity score 	0.34±0.29	*(12)*	0.51±0.41	*(7)*	t(17) = −1.06, P = 0.31
Psychiatric record (21 years old)	60%	*(6/10)*	43%	*(3/7)*	*F* exact, 2 tailled: 0.64
Criminal record (21 years old)	17%	*(2/12)*	75%	*(6/8)*	*F* exact, 2 tailled: *0.019*
Self-reported violence (21 years)	10%	*(1/10)*	57%	*(4/7)*	F exact, 2 tailled: 0.10
Attention deficit score (6 to 15 years)	3.23±2.18	*(12)*	4.00±1.89	*(8)*	t(18) = −0.81, P = 0.43
Hyperactivity trajectories (6 to 15 years)	17%	*(3/12)*	50%	*(4/8)*	*F* exact, 2 tailled: 0.14
Opposition trajectories (6 to 15 years)	0%	*(0/12)*	75%	*(6/8)*	*F* exact, 2 tailled: *0.001*
Anxiety trajectories (6 to 15 years)	8%	*(1/12)*	13%	*(1/8)*	*F* exact, 2 tailled: 0.65


include mother and father occupational score, familial status (monoparental vs biparental), mother and father at birth of first child and the years of schooling of the mother and father.

### Ethics Statement

After a complete description of the study to the subjects, all participants provided written informed consent. The study was carried out in accordance with the Declaration of Helsinki, and was approved by the research ethics committee of the University of Montreal pediatric hospital (St-Justine Hospital).

### DNA methylation analysis

DNA was extracted with Wizard Genomic DNA Purification kit (Promega) from CD3+ T cells isolated from PBMC (whole mononuclear cells from peripheral blood) using CD3 dynabeads (Dynal) following the protocol from Current Protocols in Immunology (1997, sections 7.1 and 7.5.1–7.5.11). A detailed description of the methods and analyses of methylated DNA immunoprecipitation (MeDIP) and microarrays hybridization used in this study were previously described [43] and can be found in File S1. We mapped the methylation state of the promoters of nearly 20 000 genes and 400 microRNAs in triplicate using custom designed 244 K promoter tiling arrays (Agilent technologies) containing probes selected to tile ∼1000 bp upstream to ∼250 bp downstream of the transcription start site.

### Microarray analysis

Differential methylation between groups of samples was determined at the probe and promoter levels to ensure both statistical significance and biological relevance as previously described [43] and can be found in File S1. At the probe level, a modified t-statistic was computed for each probe corresponding to probe log-ratio differences between CPA and control groups using the ‘limma’ package [50] of Bioconductor [51]. Then, promoter-level methylation differences were identified as those promoters significantly enriched with probes having positive or negative t-statistics using the Wilcoxon rank-sum test. A probe and the containing promoter were called *differentially methylated* if the p-value of the probe t-statistic was at most 0.05 (uncorrected for multiple testing), log2-fold change between the groups was at least 0.25, and the false discovery rates (FDR) of the promoter-level statistic was at most 0.2.

The ‘false discovery rate’ (FDR) is used throughout the text to judge the statistical strength of our results. FDR is computed using the Benjamini-Hochberg algorithm, which is designed to control for errors due to multiple statistical tests. Our use of a false discovery rate of 0.2 as a threshold for calling differential methylation implies that we expect at most 20% of our calls to be erroneous. This approach and the threshold of 0.2 allows us to test our hypothesis that T cell DNA methylation is associated with aggression trajectory and to generate further hypotheses about the functions of genes affected by the associated methylation changes. All functional analysis was done using Ingenuity Pathway Analysis with the default parameters as it was shown to be a reliable approach to identify biological pathways that influence disease outcomes [52].

The microarray data are available at http://www.ncbi.nlm.nih.gov/geo under the accession number GSE50674.

### Microarray validation

Differentially methylated regions from the microarray analysis with varying p values (between 0.0001 and 0.03 with FDR<0.2) and fold differences (Log_2_ CPA/Controls between 1.1 and 0.5) at the probe level were selected for validation. Two different techniques were applied on the same subjects used for microarray experiments to validate the regions called differentially methylated from the microarray analysis (n = 8 CPA and n = 12 Controls); quantitative real-time PCR on the immunoprecipitated DNA samples (Q-MeDIP) and pyrosequencing of bisulfite treated DNA (see File S1 for a more detailed description and Table S4 for the list of primers used). Moreover, using a Bayesian deconvolution method to estimate methylation levels from the data [53], we found promoter methylation levels to be significantly inversely correlated (p value = 2.3e-25) with previously published gene expression levels of T cells from human samples (Figure S1). More than 60% of the genes analyzed by the microarray have inversely correlated promoter DNA methylation and expression levels (defined as gene promoters with methylation above 50% and expression below the 50-percentile of expression levels, and vice versa).

### Rationale for the MeDIP microarray hybridization approach

MeDIP was selected among many other methods for methylation profiling because it is one of the few methods that is feasible for studying genome-wide methylation differences between groups of subjects. It has been successfully applied in many published studies [53]–[73], and it has been found competitive with the other high-throughput profiling methods that are in use [74]–[80]. Further information about MeDIP and supporting statistics from this study can be found in File S1.

## Results

### T cell promoter methylation associate with chronic physical aggression

**Figure 1:**
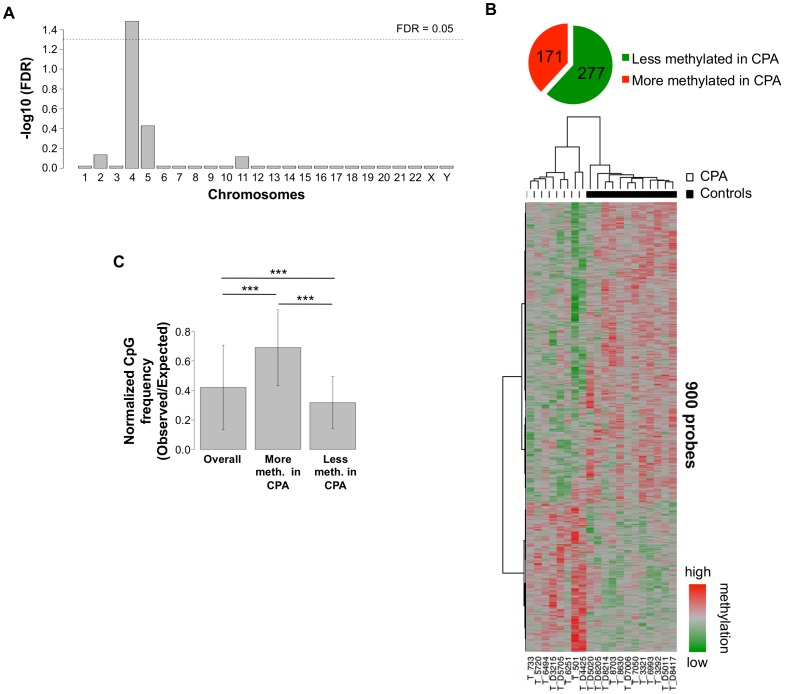
Gene promoters differentially methylated between CPA (n = 8) and controls (n = 12) in T cells. **A**. Bar heights indicate the degree to which each chromosome contains an unexpectedly high number of differentially methylated promoters. This was calculated by Fisher's exact test followed by adjustment for multiple testing by converting p-values to false discovery rates. Bar heights are -log10 values of the false discovery rates, so bars higher than the dashed line have false discovery rates below 0.05. **B**. Heatmap depicts normalized intensities of microarray probes contained in promoters (at most one per promoter) that best differentiate between CPA and control groups. Rows correspond to promoters and columns to subjects. Red indicates higher methylation in a row and green indicates lower methylation. The 900 differentially methylated probes represent 448 gene promoters where 171 are more methylated in CPA and 277 less methylated. **C**. CpG density of the differentially methylated promoters. Gene promoters more methylated in CPA have higher CpG density than the average CpG content of all promoters analyzed (p<6.3E-27). In contrast, promoters less methylated in CPA have lower CpG density than the overall average (p<0.00014). Gene promoters more methylated in CPA have also higher CpG density than the promoters less methylated in CPA (p<1.3E-33; Wilcoxon rank-sum test). Normalized CpG density is the density of CpG sites divided by the expected density calculated by multiplying the density of C sites by the density of G sites.

The methylation levels of hundreds of promoters scattered across the genome were found to be associated with CPA with statistically significant overrepresentations of associations in chromosomes 4 and 5 (p ≤ 0.01; hypergeometric, Figure 1A). In all, we found 900 probes from 448 distinct gene promoters whose normalized intensities were significantly associated with chronic aggressive behavior (FDR<0.2, Spreadsheet 1). Of these promoters, 277 were more methylated in the control group, and 171 were more methylated in the CPA group. This differentially methylated list of promoters includes 2 microRNA promoters that are more methylated in the CPA group and 10 microRNA promoters that are less methylated in the CPA group (out of 400 included on the microarray). A heatmap of the probes in each of these gene promoters that best differentiate between the groups is shown in Figure 1B. When applying a more stringent criteria to our list of differentially methylated promoters, such as FDR<0.05, 184 probes located within 60 gene promoters remained significantly associated with CPA (Spreadsheet S1). We have further confirmed methylation differences at several sites using two different techniques, Q-MeDIP (Figures S2A and S2B) and pyrosequencing (Figure S2C) as described in the Methods section.

### Functional relevance of chronic physical aggression associated with T cells methylation

If changes in methylation play a regulatory role in T cells, they should appear in the regulatory regions of genes that are actively expressed in T cells. Using a publicly available gene expression dataset of 79 different cell types, we found that most of the differentially methylated genes are indeed expressed in T cells (see File S1). Interestingly, the list of genes that are differentially methylated in CPA includes five genes that were previously linked with aggression (Table 2). *AVPR1A*, *HTR1D* and *GRM5* are less methylated in the CPA group and *DRD1* and *SLC6A3* genes are more methylated in the CPA group.

**Table 2:** Differentially methylated genes previously shown to be associated with aggressive behavior.

Gene ID	P value	FDR	Methylation	Association	Species	Type of aggression	Effects	References
***GRM5***	0.00001	0.01	less in CPA	Receptor activity (antagonist)	Mouse	Offensive aggression	Suppressed	*Navarro et al., 2006*
***DRD1***	0.005	0.08	more in CPA	Genetic (SNP)	Dog	Human-directed canine aggression	Increased	*Vage et al., 2010*
				Genetic (SNP)	Human	Physical aggression	Increased	*Sweet et al., 1998*
***AVPR1A***	0.03	0.05	less in CPA	Receptor activity (antagonist)	Hamster	Offensive aggression	Suppressed	*Ferris et al., 2006; Ferris and Potegal, 1988*
				Genetic (Microsatellite)	Prairie vole	Offensive aggression	Increased	*Hammock et al., 2005*
***HTR1D***	0.04	0.05	less in CPA	Genetic (SNP)	Dog	Human-directed canine aggression	Increased	*Vage et al., 2010*
***SLC6A3***	0.04	0.06	more in CPA	Genetic (VNTR)	Human (adolescents and young adults)	Violent delinquency	Increased	*Guo et al., 2007*
				Genetic (VNTR)	Human (adolescents)	Chronic criminal and dangerous behavior	Increased	*Vaughn et al., 2009*

We also used the Ingenuity Pathway Analysis software to determine which, if any, gene functions were significantly enriched with genes whose differential promoter methylation levels were associated with chronic aggression. The most enriched functional categories with genes whose promoters were less methylated in the CPA group included behaviour (hyperactivity), metabolic diseases (adiposity) and inflammatory response (chemotaxis of phagocytes). In contrast, the most enriched functional categories with genes whose promoters were more methylated in the CPA group included neurological diseases (encephalopathy), cellular growth and proliferation (cancer and blood cells) and gene expression (Table S1). Specific canonical pathways enriched with such genes included PPAR signalling, cytokine signaling between immune cells and G-protein coupled receptor signalling (Table S2). Furthermore, IPA allows testing for significant enrichment of targets of specific transcription factors. Thirty-four transcription factors were identified to have a significant overlap with our list of affected genes with *STAT6*, *SWI-SNF* and *FOXH1* being at the top of the list (Table S3). STAT6 is part of the STAT family of transcription factors, its phosphorylation in response to cytokines and growth factors activates the transcription of many genes involved in the immune system such as interleukin 4 (IL-4). Previous studies have shown that STAT6-deficient mice are more hyperactive and have lower levels of midbrain dopamine transporter [77] suggesting that STAT6 is involved in behavior [81].

### Differentially methylated promoters in CPA have distinct CpG densities

CpG islands are small regions with unusually high CpG densities that are typically unmethylated [82], particularly when found near the transcription start sites of genes [82]. We observed extremely high CpG densities in promoters with higher methylation in the CPA group in contrast to unusually low CpG densities in promoters with lower methylation in the CPA group (Figure 1C). We note that it was previously shown that genes with high CpG densities in their promoters tend to be involved in housekeeping activities in the cell [83].

### Methylation associated with chronic physical aggression clusters by genomic location

**Figure 2:**
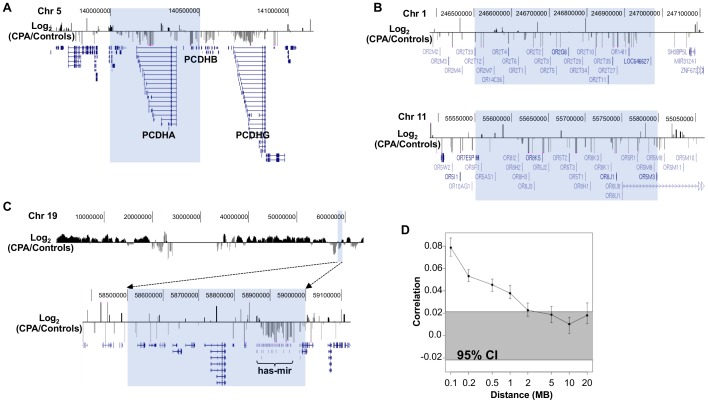
Megabase co-clustering of differential methylation between CPA (n = 8) and controls (n = 12). **A**. Co-clustering of differential methylation among the protocadherins genes. Positive values (black bars) indicate increased methylation in CPA compared to controls and negative values (grey bars) indicate the opposite. Shaded in blue is a 500 Kb region containing protocadherins family A and B whose promoters are consistently less methylated in CPA than in controls (scale log2 fold differences: −0.2 to 0.2). **B**. Co-clustering of differentially methylated promoters with common function across megabases of DNA. The olfactory receptor clusters located on chromosome 1 and chromosome 11 are less methylated in CPA compared to controls (scale log2 fold differences: −0.2 to 0.2). **C**. On chromosome 19, one of the few megabase regions showing decreased methylation in CPA compared to controls contains one of the two human micro-RNA clusters (scale log2 fold differences top and bottom panels: −0.2 to 0.2). **D**. Methylation dependences across megabases are shown. Pearson correlations of DNA methylation differences between controls and CPA groups at various genomic distances are shown. Error bars show 95% confidence intervals for the correlation values. The grey highlight shows the expected 95% confidence interval if there is no correlation between methylation differences at different genomic sites. This confidence interval does not overlap with the error bars associated with distances less than 2 Mb suggesting the existence of systematic dependencies between methylation differences at distances up to 2 Mb.

In spite of the fact that differences in DNA methylation associated with aggression groups were distributed uniformly across the chromosomes, with higher density in two chromosomes (4 and 5), probes with similar differences tended to appear in clusters within chromosomes. To measure the strength of this clustering, we partitioned the genome into 500 Kb regions and asked whether each region contained a surprisingly high number of differentially methylated probes. Of the ∼6 K regions, we found that 6 were significantly enriched for decreased methylation in the CPA group (Table 3) even after adjustment for multiple testing. Four of these six regions are located in known gene clusters: the protocadherin alpha cluster, two olfactory receptor clusters and a recently identified human microRNA cluster (Figure 2). From the 718 microRNAs that composed the cluster on chromosome 19, at least 148 (21%) are expressed in T cells according to smirnaDB, a database of microRNA expression profiles [84]. Protocadherins are a superfamily of genes that encode proteins principally involved in cell adhesion. Interestingly, differential methylation of the protocadherin cluster was also found to be associated with childhood socioeconomic position in whole blood [85] and with differential maternal care early in life in hippocampi of rats [33]. In general, methylation differences also tended to cluster across the entire genome. Beyond specific 500 Kb partitions containing clusters of differential methylation, we found that methylation differences as far apart as 2 Mb displayed a small but significant level of interdependence. The statistics are illustrated in Figure 2D and further details can be found in File S1.

**Table 3:** List of chromosomal regions differentially methylated between CPA and control groups.

Regions	Locations	FDR	Gene cluster	Gene promoters more or less methylated in CPA
**1**	Chr1: 246500001–247000001	4.75E-5	Olfactory receptors	*OR2T10*
**2**	Chr19: 58500001–59000001	2.24E-4	Has-mir	*hsa-mir-517B, hsa-mir-520D, hsa-mir-520G*
**3**	Chr11: 55500001–56000001	1.97E-2	Olfactory receptors	*OR8J1*
**4**	Chr12: 10000001–10500001	0.10		*KLRC3*
**5**	Chr5: 140000001–140500001	0.11	Protocadherins	*PCDHA5, PCDHA10, PCDHA12, PCDHB2*
**6**	Chr3: 50000001–50500001	0.17		

### Promoter methylation associated with men CPA is also observed in women CPA

**Figure 3:**
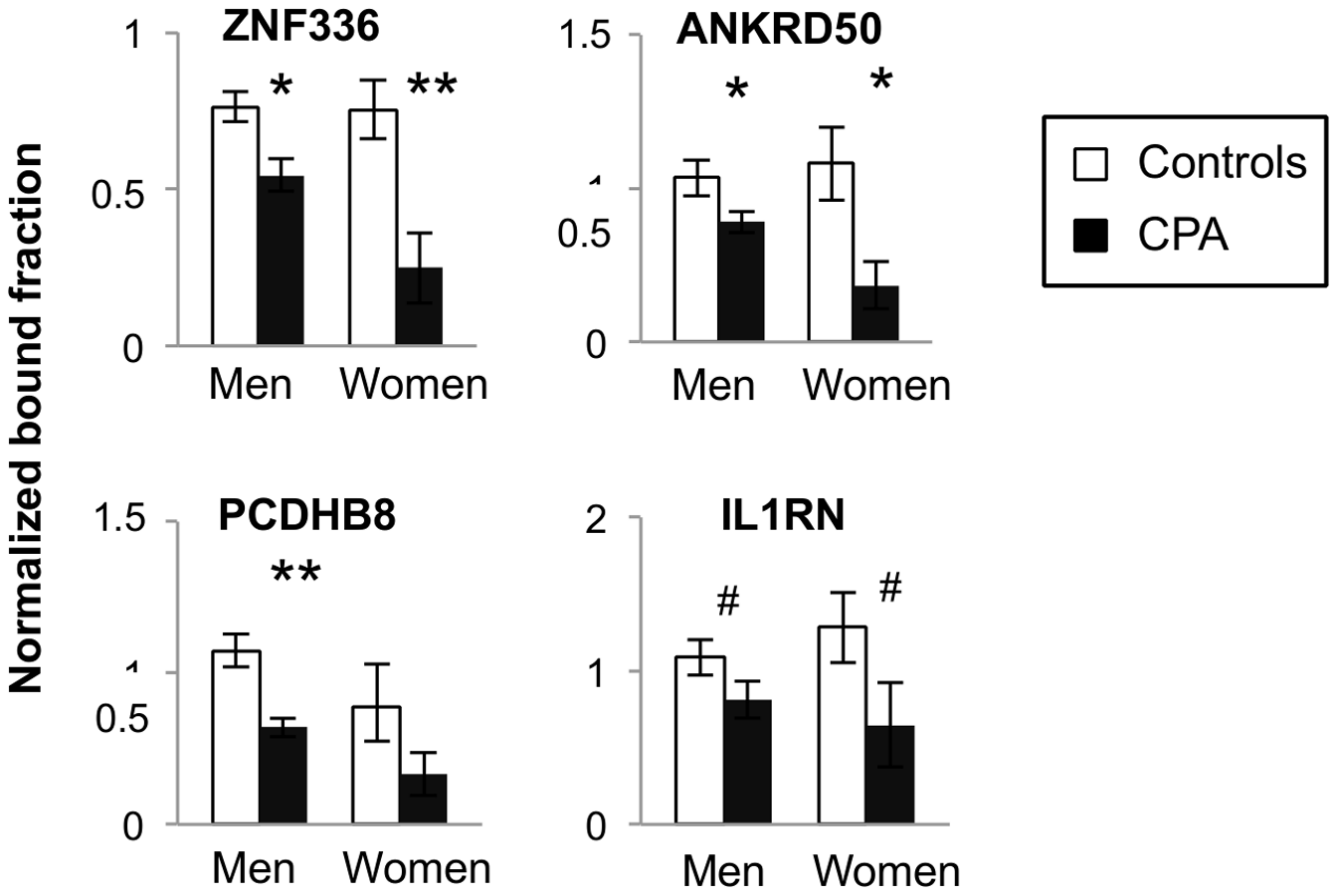
Q-MeDIP analysis in women T cells of four promoters that are differentially methylated between CPA and control groups in men. Q-MeDIP analysis of DNA methylation differences between CPA (black) and control (white) groups in men and women T cells samples for four gene promoters predicted to be more methylated in the men CPA group by microarray analysis. Relative bound fraction concentrations obtained in triplicate by Q-MeDIP are shown (see methods). All error bars represent standard error of the mean (SEM). The *P* value obtained from Mann-Whitney U test is represent by #≤0.1, *≤0.05 and **≤0.01.

In order to determine whether the methylation differences associated with CPA in men are seen in a different human sample, we used preliminary data from an ongoing study in the laboratory in women selected from the same longitudinal study as the one used here for the aggressive men study. We confirmed by qPCR of the MeDIP DNA that four promoters that are differentially methylated between CPA and control groups in T cells DNA in men are also differentially methylated in women. The Q-MeDIP analysis showed that these promoters were less methylated in the CPA group in men and in women (Figure 3). The validation of these differentially methylated promoters in a distinct human sample, and of a different sex, is consistent with the hypothesis that chronic physical aggression is associated with differential methylation in these promoters.

## Discussion

This study provides evidence of clustered and genome-wide variation in promoter DNA methylation in young adults that associates with a history of chronic physical aggression from 6 to 15 years of age. Probes with similar differences in DNA methylation between the compared groups appear in clusters within chromosomes. Indeed, we found six 500 Kb regions significantly enriched for decreased methylation in the CPA group compared to the control group (Table 3). Four of these regions are well defined gene clusters containing the olfactory receptor and the protocadherins as well as a newly-defined human microRNAs cluster previously shown to be regulated by DNA methylation in cancer cells [86].

Consistent with the fact that these differences are associated with aggression, we validated four of these differentially methylated promoters in a distinct human sample and of a different sex. Moreover, we found 5 genes amongst the 448 differentially methylated promoters that were previously shown to be involved in physical aggression in animals and in humans (Table 2). First, we found that *AVPR1A* promoter is less methylated in the CPA group, which is consistent with higher activity. Indeed, blocking AVPRIA with a specific antagonist decreases aggressive behavior in hamsters [87] while AVP the agonist of AVPRIA, enhances aggression in animals and humans [88]. Second, *DRD1* and *SLC6A3* genes have higher promoter methylation in the CPA group and increased brain dopamine levels are thought to be positively associated with aggression [20], [89]. A genetic association was found in the gene coding for *DRD1* with psychosis and aggression in Alzheimer patients [90]. Moreover, a variable number of tandem repeat in the 3′UTR of the *SLC6A3* gene is associated with various antisocial behaviors such as violent delinquency [91] and propensity to a criminal career [92]. Third, *GRM5* promoter is less methylated in CPA and Navarro et al. showed that 2-methyl-6-(phenylethylnyl)pyridine (MPEP), a selective antagonist of GRM5, has anti-aggressive effects in mice [93]. Elevated glutamatergic activity in the brain has been associated with aggression in animals and human [94]. Finally, the inhibitory autoreceptor *HTR1D* promoter is less methylated in CPA and lower serotonin levels in the brain and the periphery associate with high aggression in animals and humans [95]. Allelic association with aggression in dogs was observed in the gene coding for the receptor *HTR1D* [96]. Since genetic associations with aggression were observed for *AVPR1A*, *HTR1D*, *DRD1* and *SLC6A3* (Table 2), it is possible that there is an interaction between genetic variations and differential DNA methylation in aggression. Recent studies have found such interactions in asthma [97], chronic fatigue syndrome [98], trauma [99], and gastric cancer [100]. In our samples, the small number of subjects did not allow for testing of genetic association and their interactions with DNA methylation.

It is noteworthy that although these genes are clearly involved in brain function, we observed changes in DNA methylation associated with aggressive behavior in T cells. We obviously don't know whether similar changes occur in the brain of the same subjects. Nevertheless, these observations are consistent with the notion that T cells will be informative not only on immune specific genes that are associated with the HPA axis but also on some genes that are also involved in brain function. A parallel comparison of DNA methylation changes in prefrontal cortex and T cells in response to differential rearing conditions in rhesus monkeys revealed both tissue specific alterations as well as common differentially methylated regions in T cells and prefrontal cortex [101]. We also reported recently that the DNA methylation state of the *SLC6A4* promoter in T cells and monocytes inversely associates with positron emission tomography (PET) measures of brain serotonin synthesis and that both measures associate with aggression in humans [46]. However, only a parallel investigation of T cells and brain like the one that we performed in non-human primates could confirm whether specific DNA methylation changes occur in the brain as well.

As anticipated, since we are working with blood samples, the inflammatory and immune response categories with specific signaling pathway such as cytokines signaling between immune cells, IL-6 and IL-10 signaling were identified in our analysis. Indeed, several lines of evidence suggest that cytokines are associated with animal and human aggression [102]–[104] and IL-6 was causally linked to aggression in mice by gene knockout evidence [105]. Specific cytokines and receptors involved in these pathways were previously shown to be involved in aggression and human mood disorders. IL1R1 and IL1RN have been shown to be involved in defensive aggression through their activation by IL-1β [106], [107]. Moreover, our previous research done on the same men as the one studied here, revealed an association between cytokine expression and methylation with chronic physical aggression during childhood [47], [48]. Further work is needed to investigate the exact role of these molecules in the development of chronic aggression, but taken together these data are consistent with the hypothesis that cytokine regulation could be involved in human behavior and behavioral disorders.

The changes in DNA methylation that we observe between CPA and controls are numerous and significant but each individual effect is small (i.e. per-probe fold change is small). Although it is possible to brush off these subtle changes as biologically irrelevant, their consistency and statistical significance point to the possibility of an important biological role whereby the epigenome is modulated by a combination of small changes across functional pathways and chromosomal regions. It is important to note in this respect that DNA methylation is a binary signal, that is, a site is either methylated or unmethylated in a given cell. Therefore a partial methylation such as is observed in our study indicates that a small but statistically significant subpopulation of cells is differentially methylated. A challenge for future experiments is to identify the cellular populations that exhibit these changes in DNA methylation and to understand their biological role.

Although the present study is the first to show an association between chronic physical aggression, and differential DNA methylation, there are several methodological limitations. First, because DNA was available only in adulthood, we cannot establish when chronic physical aggression became associated with the observed methylation patterns and whether they precede or follow the appearance of the aggressive phenotype. To sort out the sequence of events with human samples we will need longitudinal data on DNA methylation from birth and physical aggression from infancy onwards. Second, we do not know the extent to which our observed association between methylation and aggression carries over into the brain. Third, the study was limited by the lack of good quality RNA since we were unable to have the chronically aggressive subjects come to the lab for their blood draw. Fourth, there was no psychometric-physical evaluation at the time of blood draw. The acute psychological and/or physical status might confound our findings. In this respect, longitudinal data on the T-cell methylomes would have been highly valuable but the original study design didn't include brood draws at multiple time points. Future longitudinal studies that include concurrent blood draws and psychometric-physical evaluations are required to address this question.

Taken together, the findings of differentially methylated genes relevant to CPA and clustering of CPA associated methylation across the genome suggests a well-defined, genome-wide epigenetic pattern associated with chronic physical aggression in humans.

## Supporting Information

Figure S1 **Distributions of promoter methylation levels by published expression level in T cells.** Genes are divided into 20 levels by T cells expression percentiles (0–5, 5–10,…, 95–100) based on publicly available expression data(10). Shown are the distributions of methylation levels for each expression percentile. The distributions show that genes with low or no expression (represented in green) tend to have highly methylated promoters, whereas genes with high expression (represented in red) tend to have lower promoter methylation.
(TIF)

Figure S2 **Microarray validation by Q-MeDIP and pyrosequencing of differentially methylated sequences between CPA (n = 8) and control groups (n = 12).** **A.** Fold differences between CPA and control groups obtained by either Q-MeDIP or microarray analysis are shown for 20 genes predicted to be either more methylated (n = 1) or less methylated (n = 19) in the CPA individuals by the microarray analysis. **B**. Correlation of the fold differences between CPA and control obtained by Q-MeDIP and by microarray for the 20 amplicons analyzed in A. **C**. DNA methylation differences (%) between the groups in the *GRM5, ITGB6, OR13C8* and *IL-31* genes validated by pyrosequencing. CpG sites near the significant probe were analyzed. For each gene, the mean methylation per aggressive group per CpG is shown in the bar graph. The rightmost bar indicates the mean methylation levels of all CpG sites analyzed in the region. A map of the sites relative to the transcription start site is shown above the bar graph. Each line represents a CpG site. The location of probes whose fold difference is significantly different between CPA and control groups is identified by a grey square. The region analyzed by pyrosequencing is delimited by the red arrows. All error bars represent standard error of the mean (SEM). The *P* value obtain from Mann-Whitney U test is represent by *≤0.05, **≤0.01 and ***≤0.001.
(TIF)

File S1 **Supplementary information file containing additional information of the methods use in the study.**
(DOCX)

Spreadsheet S1 **List of the probes differentially methylated between the chronic physical aggression and the control groups.**
(XLSX)

Table S1 **Top list of affected biological functions enriched with genes whose methylation is associated with aggression from Ingenuity Pathway (12) analysis (n = 448 genes).** All of the p values were calculated using a right tailed Fisher's exact test and corrected for multiple comparison with the Benjamini-Hochberg method. Significance threshold were p = 0.05.
(DOCX)

Table S2 **Top list of canonical pathways enriched with genes whose methylation is associated with aggression from IPA analysis (n = 448 genes).** All of the p values were calculated using a right tailed Fisher's exact test and corrected for multiple comparison with the Benjamini-Hochberg method. Significance threshold were p = 0.05.
(DOCX)

Table S3 **List of transcription regulators showing a significant overlap with genes whose methylation is associated with aggression from IPA analysis (n = 448 genes).** Transcription regulators differentially methylated between chronic and normal aggression are shown in bold. Significance threshold were p = 0.05.
(DOCX)

Table S4 **Primers and melting temperature (Tm) used for Q-MeDIP and pyrosequencing.**
(DOCX)
