# Supplementary figures and images for: Association of Childhood Chronic Physical Aggression with a DNA Methylation Signature in Adult Human T Cells
Source: PLoS One. 2014 Apr 1;9(4):e89839. doi: 10.1371/journal.pone.0089839 (PMC3972178; doi:10.1371/journal.pone.0089839)

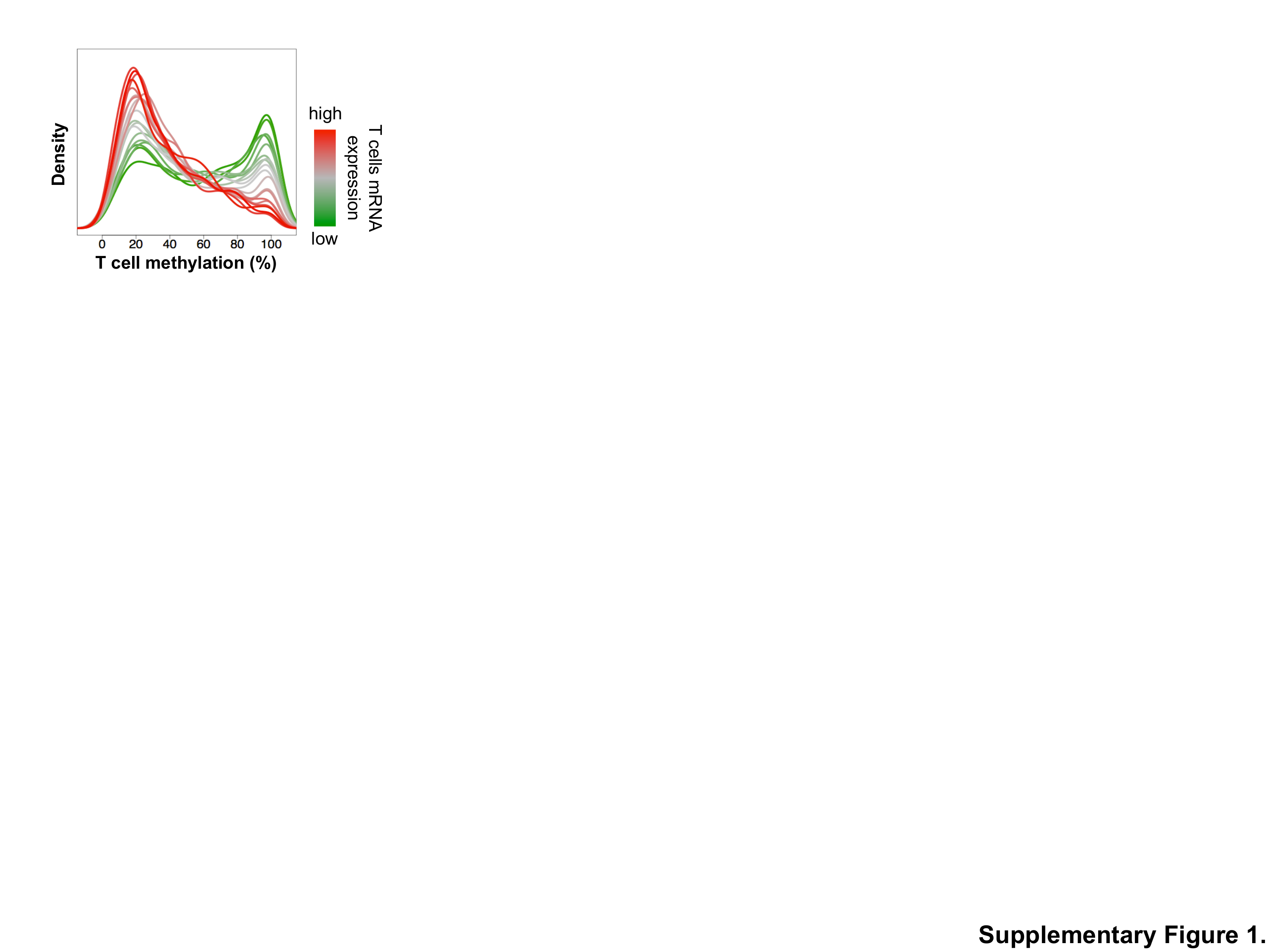

Supplement: Figure S1 — Distributions of promoter methylation levels by published expression level in T cells. Genes are divided into 20 levels by T cells expression percentiles (0–5, 5–10,…, 95–100) based on publicly available expression data(10). Shown are the distributions of methylation levels for each expression percentile. The distributions show that genes with low or no expression (represented in green) tend to have highly methylated promoters, whereas genes with high expression (represented in red) tend to have lower promoter methylation. (TIF) [file pone.0089839.s001.tif]

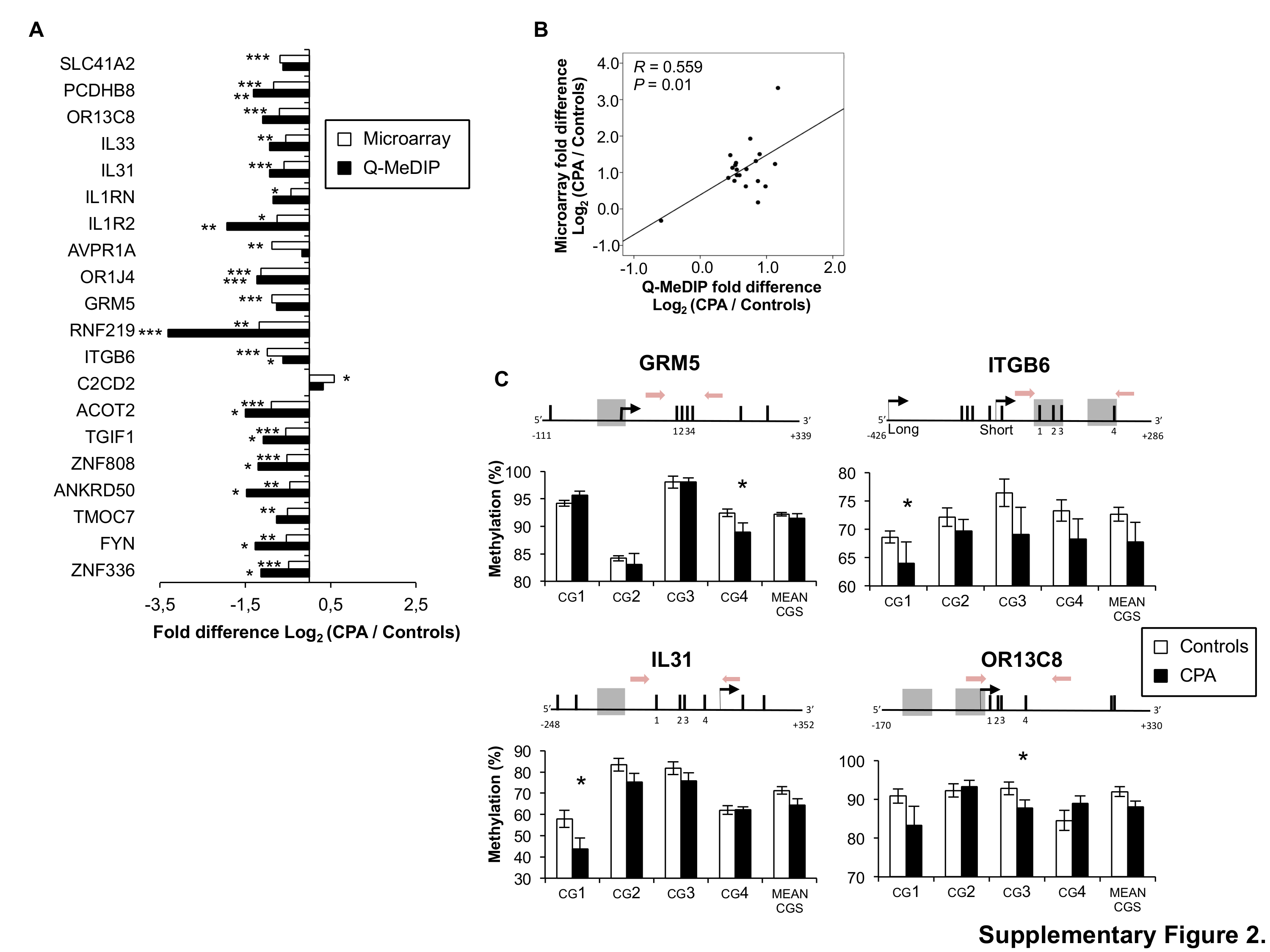

Supplement: Figure S2 — Microarray validation by Q-MeDIP and pyrosequencing of differentially methylated sequences between CPA (n = 8) and control groups (n = 12). A. Fold differences between CPA and control groups obtained by either Q-MeDIP or microarray analysis are shown for 20 genes predicted to be either more methylated (n = 1) or less methylated (n = 19) in the CPA individuals by the microarray analysis. B. Correlation of the fold differences between CPA and control obtained by Q-MeDIP and by microarray for the 20 amplicons analyzed in A. C. DNA methylation differences (%) between the groups in the GRM5, ITGB6, OR13C8 and IL-31 genes validated by pyrosequencing. CpG sites near the significant probe were analyzed. For each gene, the mean methylation per aggressive group per CpG is shown in the bar graph. The rightmost bar indicates the mean methylation levels of all CpG sites analyzed in the region. A map of the sites relative to the transcription start site is shown above the bar graph. Each line represents a CpG site. The location of probes whose fold difference is significantly different between CPA and control groups is identified by a grey square. The region analyzed by pyrosequencing is delimited by the red arrows. All error bars represent standard error of the mean (SEM). The P value obtain from Mann-Whitney U test is represent by *≤0.05, **≤0.01 and ***≤0.001. (TIF) [file pone.0089839.s002.tif]
