# Supplementary material for: Association of Childhood Chronic Physical Aggression with a DNA Methylation Signature in Adult Human T Cells
Source: PLoS One. 2014 Apr 1;9(4):e89839. doi: 10.1371/journal.pone.0089839 (PMC3972178; doi:10.1371/journal.pone.0089839)
Supplement: File S1 — Supplementary information file containing additional information of the methods use in the study. (DOCX) [file pone.0089839.s003.docx]

**Supplementary Information**

**Supplementary Materials and Methods**

**Assessment of subjects’ characteristics.**

*Familial adversity*. The index of family adversity is a composite score of the degree of adversity in families ranging from 0 to 1, which has been used regularly with these cohorts and is describe in details elsewhere ([1](#_ENREF_1), [2](#_ENREF_2)). The index includes parent’s level of education, type of employment, age at birth of their first child and marital status when the subjects were age 6 ([1](#_ENREF_1), [2](#_ENREF_2)).

*Physical aggression and other behavior problems.* In the course of the longitudinal studies, teachers annually rated the frequency of the boys’ physical aggression, opposition, hyperactivity, inattention and anxiety from kindergarten to secondary school with the Social Behavior Questionnaire([1](#_ENREF_1)). The physical aggression ratings were used to trace the developmental trajectories and create the CPA group and the control group (see Broidy et al., 2003 and Nagin & Tremblay, 1999 for details of the trajectory analyses). The same approach was used to create the chronic and non-chronic groups using the developmental trajectories for hyperactivity, opposition and anxiety (see Nagin & Tremblay, 1999 for details of the trajectory analyses). The aggressive groups (CPA and controls) were compared to see if one was enriched with subjects on the chronic trajectories for hyperactivity, opposition and anxiety variables. The inattention deficit score was calculated by computing the mean of the score obtained at each year when the boys were from 6 to 15 years old.

*Self-reported violence.* During the data collection at 21 years, subjects were asked how often in the past year they had been implicated in physical fights and how often they had physically attacked someone.

*Criminal record.* Canadian youth between 13 and 17 years who commit delinquent acts are referred to the juvenile courts. From official records we identified subjects who were found guilty by a juvenile court. From official records we also identified subjects in each group who had been convicted of a criminal offence between 18 and 24 years.

*Mental disorders.* When the subjects were 15 years old, structured psychiatric interviews using a French translation of the Diagnostic Interview Schedule for Children-2.25 (DISC-2.25) were used with the mothers and the subjects to estimate the prevalence of DSM-III-R diagnoses such as: simple phobia, anxiety of separation, generalized anxiety, hyper anxiety, major depression, dysthymia, oppositional disorder and conduct disorder, over the previous 6 months ([3](#_ENREF_3)). Subjects were also asked if they had a psychiatric record during the interview at 21 years. A total of 9 subjects have been diagnosed either at 15 or stated to have a psychiatric record at 21 years old, three CPA and six controls. At 15 years old, the following diagnosis were identified for 5 of these 9 subjects: one CPA had conduct disorder; one CPA had conduct disorder and hyper anxiety; one control had simple phobia; one control had simple phobia and conduct disorder and one control had generalized anxiety, dysthymia and conduct disorder.

**CD3+ T cell DNA preparation.** For the study, 20 ml of blood was drawn in EDTA coated-tubes and store at 4°C until cells isolation (max 24 hours). PBMC (whole mononuclear cells from peripheral blood) and T cell isolation procedures were adapted from Current Protocols in Immunology (1997, sections 7.1 and 7.5.1-7.5.11). Briefly, PBMC isolation was done by centrifugation with Ficoll-Paque (GE healthcare) and washed twice with HBSS (Hanks balanced salt solution, GIBCO). T cells were isolated from the PBMCs by immunomagnetic isolation using CD3 dynabeads (Dynal). The beads were washed 3 times and incubated with the PBMCs for 45 min on a rotator at 4°C. Coated CD3+ cells with the dynabeads were isolated using a strong magnet (Steam Cell Technology) and washed 5 times with PBS/FBS. CD3+ cells coated with the dynabeads were then frozen at -80°C until DNA extraction. T cells DNA was extracted with Wizard® Genomic DNA Purification kit (Promega) following the manufacturer’s protocol.

**Methylated DNA Immunoprecipitation (MeDIP), amplification and labeling.** The MeDIP analysis was adapted from([4](#_ENREF_4)). Briefly, 2 µg of each of the T cells DNA was sonicated and methylated DNA was immunoprecipitated with 10 µg of anti-5methylCytosine (Calbiochem). Prior to sonication, two control plasmids were added to the DNA (6ρg each), an unmethylated GFP plasmid and an *in vitro* methylated Luciferase plasmid. The DNA-antibody complex was immunoprecipitated with 5 mg protein A and the methylated DNA was eluted with 150 µl of TE at 1.5% SDS. The input and bound fractions were then purified and validated by PCR analysis for two control genes, H19 (methylated control) and β-actin (unmethylated control) and the two added plasmid, GFP (unmethylated control) and Luciferase (*in vitro* methylated control) using the following primers: H19: forward (5’-TTGGTGGAACACGCTGTGATCA-3’), reverse (5’-GAGCCGCACCAGGTCTTCAG-3’); β-actin: forward (5’-AGCCATAAAAGGCAACTTTCG-3’), reverse (5’-CCAACGCCAAAACTCTCCC-3’); GFP: forward (CCAACGCCAAAACTCTCCC), reverse (AGCCATAAAAGGCAACTTTCG); Luciferase: forward (5’-AGAGATACGCCCTGGTTCC-3’), reverse (5’-CCAACACCGGCATAAAGAA-3’). The input and bound fractions were then amplified in triplicate using the Whole Genome Amplification kit (Sigma). The amplified input and bound fractions were labeled for microarray hybridization with either Cy3-dUTP or Cy5-dUTP (Perkin Elmer) respectively using the CGH labeling kit (Invitrogen) following the manufacturer’s instructions.

**Microarray design, hybridization, scanning and analysis.** A detailed description of the methods and analysis concerning microarrays used in this study were previously described([5](#_ENREF_5)). Briefly, custom 244K promoter tiling arrays (Agilent technologies) containing probes selected to tile all known gene promoters were used. Three replicate microarrays were hybridized and analyzed for each sample. Extracted probe intensities were analyzed using the R software environment for statistical computing([6](#_ENREF_6)). Log-ratios of the bound (Cy5) and input (Cy3) microarray channel intensities were computed for each microarray and then microarrays were normalized to one another using quantile-normalization([7](#_ENREF_7)) under the assumption that all samples have identical overall methylation levels.

Differential methylation between groups of samples was determined at the probe and promoter levels to ensure both statistical significance and biological relevance as previously described([5](#_ENREF_5)). At the probe level, a modified t-statistic was computed for each probe corresponding to probe log-ratio differences between CPA and control groups using the ‘limma’ package([8](#_ENREF_8)) of Bioconductor([9](#_ENREF_9)). Then, promoter-level methylation differences were calculated as enrichment of large positive or negative t-statistic values among the probes in the promoter (about 10) using the Wilcoxon rank-sum test. A probe and the containing promoter were called *differentially methylated* if the p-value of the probe t-statistic was at most 0.05 (uncorrected for multiple testing), log2-fold change between the groups was at least 0.25, and the false discovery rates (FDR) of the promoter-level statistic was at most 0.2.

Figure 1B depicts heatmaps of probe intensities. Probes were selected from gene promoters called differentially methylated with respect to aggressive groups, as described above. One probe was selected for each gene, always the probe with the most extreme t-statistic. Heatmaps are colored so the median values on each row are gray, high values are red and low values are green. Clustering was performed using Ward's hierarchical clustering algorithm with Pearson correlation distance as the distance metric.

In Figure S1, gene expression levels were obtained from publicly available T cells expression profiles([10](#_ENREF_10)) that we used to partition genes by expression percentiles (0-5, 5-10, ... , 95-100). To obtain gene promoter methylation levels, we computed the average normalized intensity of each probe across all samples and replicates, and then applied the Bayesian deconvolution algorithm mentioned above to the resulting averages([11](#_ENREF_11)).

To identify 500Kb partitions of the genome with statistically significant methylation differences between aggression groups, each promoter was first assigned the probe with the most extreme t-statistic computed as described above using the “limma” package. The Wilcoxon rank-sum test was then used to compare the promoter probe t-statistics within each 500Kb partition to those outside the 500Kb partition. False positive errors due to multiple testing were controlled by calculating false discovery rates and requiring positive calls to have a have false discovery rate less than 0.2.

Figure 2D illustrates the correlation of methylation differences across various genomic distances as Pearson correlations of modified t-statistics computed by limma for all pairs of probes at specified distances (with a 10% tolerance). Error bars denote 95% confidence intervals obtained from 1000 bootstraps composed of randomly selected probe pairs with replacement. The gray rectangle denotes the 95% confidence intervals for correlations of probe pairs independent of their distance. Independence was simulated by with 500 random permutations of the probe coordinates.

All functional analysis was done using Ingenuity Pathway Analysis ([12](#_ENREF_12)) with the default parameters as it was shown to be a reliable approach to identify biological pathways that influence disease outcomes([13](#_ENREF_13)).

**Transcriptional activity of differentially methylated genes in T cells.** We compared the expression levels of our differentially methylated genes in T cells to their expression levels in 77 other cell types using a publicly available gene expression atlas([10](#_ENREF_10)). Of the 882 probes found differentially methylated, we were able to link 737 to genes with expression profiles in the gene atlas. Of these, 561 probes were associated with genes whose expression levels in T cells were higher than the median expression levels for that gene across the other 77 other cell types. In fact, this set of genes was enriched for genes with higher overall expression in T cells (p < 1.6 x 10^-16^; Wilcoxon rank-sum test).

**Microarray validation***. Quantitative real-time PCR of immunoprecipitated DNA samples (Q-MeDIP).* Gene-specific real-time PCR validation of microarray was performed on the amplified bound fraction for the same subjects used for microarray experiments (n=8 CPA and n=12 Controls). Relative enrichment of triplicate reactions were determined as a ratio of the crossing point threshold (Ct) of the amplified specific gene over the Ct of the amplified methylated control according to the formula Bound gene (Ct)/Bound control (Ct). The methylated control used is the *in vitro* methylated luciferase plasmid that was added in equal amount to each sample prior to the MeDIP. The Q-MeDIP analyses of the methylated control show no difference between the groups.

*Bisulfite treatment and pyrosequencing.*1 µg of EcoRI digested DNA was subjected to bisulfite treatment as previously described([14](#_ENREF_14)). Loci were selected for further analysis from the locations of probes whose normalized intensities were significantly different between the groups (see Microarray Analysis). PCR amplifications were performed in two steps: 25 ng of bisulfite DNA were used for the outside PCR and 1 μl of this DNA for the nested PCR. The outside and nested PCR were done using HotStarTaq DNA polymerase (Qiagen). For pyrosequencing analysis, 25 μl of the nested bisulfite-PCR products were processed according to the manufacturer's standard protocol (Biotage). Sequencing reactions were performed with a PyroMark Gold Q24 Reagent Kit (Biotage, Qiagen) according to the manufacturer's instructions. The percentage methylation at each CpG site was calculated from the raw data by use of PyroMark Q24-CpG Software (Biotage).

For Q-MeDIP validation, 20 regions were tested where 15 positively validated the microarray differentially methylated probes (11 at p < 0.05 and 4 at p < 0.1) and the remaining regions analyzed did not reach the significance threshold (Figures S2A and S2B). Eight genes were analyzed by pyrosequencing where six of them positively validated the microarray differences between the groups (4 at p < 0.05 shown in the Figure S2C, and 2 at p < 0.1) and the two other genes did not reach the significance threshold.

The full list of primers used in this study can be found in table S4.

**Rationale for the MeDIP microarray hybridization approach.** MeDIP was selected among many other methods for methylation profiling because it is one of the few methods that is feasible for studying genome-wide methylation differences between groups of subjects, it has been successfully applied in many published studies([4](#_ENREF_4), [11](#_ENREF_11), [15-33](#_ENREF_15)), and it has been found competitive with the other high-throughput profiling methods that are in use([34-40](#_ENREF_34)). Moreover, MeDIP detects DNA methylation exclusively from hydroxymethylation and is not confounded by chemical conversion and biased amplification of converted sequences. Indeed, the MeDIP profiles created for this study met our expectations. Firstly, technical replicates have higher association with one another than with those from other samples, such that according to the eigenR2 algorithm([41](#_ENREF_41)), differences between samples account for 38% of the variation in the data. Secondly, using a Bayesian deconvolution method to estimate methylation levels from the data([11](#_ENREF_11)), we found promoter methylation levels to be significantly anti-correlated (p value=2.3e-25) with previously published gene expression levels of the same cell type from human samples (Figure S1). Finally, methylation differences obtained using a highly conservative approach based on linear models were similarly validated using both PCR and bisulfite sequencing-based approaches and even replicated on a separate sample.

**References**

1. Tremblay RE, Loeber R, Gagnon C, Charlebois P, Larivee S, LeBlanc M. Disruptive boys with stable and unstable high fighting behavior patterns during junior elementary school. J Abnorm Child Psychol. 1991; 19(3): 285-300.

2. Tremblay RE, Nagin DS, Seguin JR, Zoccolillo M, Zelazo PD, Boivin M, et al. Physical aggression during early childhood: trajectories and predictors. Pediatrics. 2004; 114(1): e43-50.

3. Romano E, Tremblay RE, Vitaro F, Zoccolillo M, Pagani L. Prevalence of psychiatric diagnoses and the role of perceived impairment: findings from an adolescent community sample. J Child Psychol Psychiatry. 2001; 42(4): 451-61.

4. Keshet I, Schlesinger Y, Farkash S, Rand E, Hecht M, Segal E, et al. Evidence for an instructive mechanism of de novo methylation in cancer cells. Nat Genet. 2006; 38(2): 149-53.

5. Borghol N, Suderman M, McArdle W, Racine A, Hallett M, Pembrey M, et al. Associations with early-life socio-economic position in adult DNA methylation. Int J Epidemiol. 2012; 41(1): 62-74.

6. R Development Core Team. R: A language and environment for statistical computing. R Foundation for Statistical Computing, 2007.

7. Bolstad BM, Irizarry RA, Astrand M, Speed TP. A comparison of normalization methods for high density oligonucleotide array data based on variance and bias. Bioinformatics. 2003; 19(2): 185-93.

8. Smyth GK. Limma: linear models for microarray data. In: Bioinformatics and Computational Biology Solutions using R and Bioconductor (ed VC R. Gentleman, S. Dudoit, R. Irizarry, W. Huber ): 397-420. Springer,, 2005.

9. Gentleman RC, Carey VJ, Bates DM, Bolstad B, Dettling M, Dudoit S, et al. Bioconductor: open software development for computational biology and bioinformatics. Genome Biol. 2004; 5(10): R80.

10. Su AI, Wiltshire T, Batalov S, Lapp H, Ching KA, Block D, et al. A gene atlas of the mouse and human protein-encoding transcriptomes. Proc Natl Acad Sci U S A. 2004; 101(16): 6062-7.

11. Down TA, Rakyan VK, Turner DJ, Flicek P, Li H, Kulesha E, et al. A Bayesian deconvolution strategy for immunoprecipitation-based DNA methylome analysis. Nat Biotechnol. 2008; 26(7): 779-85.

12. Rossi RL, Rossetti G, Wenandy L, Curti S, Ripamonti A, Bonnal RJ, et al. Distinct microRNA signatures in human lymphocyte subsets and enforcement of the naive state in CD4+ T cells by the microRNA miR-125b. Nat Immunol. 2011; 12(8): 796-803.

13. Ngwa JS, Manning AK, Grimsby JL, Lu C, Zhuang WV, Destefano AL. Pathway analysis following association study. BMC Proc. 2011; 5 Suppl 9: S18.

14. Clark SJ, Harrison J, Paul CL, Frommer M. High sensitivity mapping of methylated cytosines. Nucleic Acids Res. 1994; 22(15): 2990-7.

15. Bell CG, Finer S, Lindgren CM, Wilson GA, Rakyan VK, Teschendorff AE, et al. Integrated genetic and epigenetic analysis identifies haplotype-specific methylation in the FTO type 2 diabetes and obesity susceptibility locus. PLoS One. 2010; 5(11): e14040.

16. Cheung HH, Lee TL, Davis AJ, Taft DH, Rennert OM, Chan WY. Genome-wide DNA methylation profiling reveals novel epigenetically regulated genes and non-coding RNAs in human testicular cancer. Br J Cancer. 2010; 102(2): 419-27.

17. Flanagan JM, Cocciardi S, Waddell N, Johnstone CN, Marsh A, Henderson S, et al. DNA methylome of familial breast cancer identifies distinct profiles defined by mutation status. Am J Hum Genet. 2010; 86(3): 420-33.

18. Guerrero-Bosagna C, Settles M, Lucker B, Skinner MK. Epigenetic transgenerational actions of vinclozolin on promoter regions of the sperm epigenome. PLoS One. 2010; 5(9).

19. Gunther T, Grundhoff A. The epigenetic landscape of latent Kaposi sarcoma-associated herpesvirus genomes. PLoS Pathog. 2010; 6(6): e1000935.

20. Hiura H, Sugawara A, Ogawa H, John RM, Miyauchi N, Miyanari Y, et al. A tripartite paternally methylated region within the Gpr1-Zdbf2 imprinted domain on mouse chromosome 1 identified by meDIP-on-chip. Nucleic Acids Res. 2010; 38(15): 4929-45.

21. Lempiainen H, Muller A, Brasa S, Teo SS, Roloff TC, Morawiec L, et al. Phenobarbital mediates an epigenetic switch at the constitutive androstane receptor (CAR) target gene Cyp2b10 in the liver of B6C3F1 mice. PLoS One. 2011; 6(3): e18216.

22. Morris MR, Ricketts CJ, Gentle D, McRonald F, Carli N, Khalili H, et al. Genome-wide methylation analysis identifies epigenetically inactivated candidate tumour suppressor genes in renal cell carcinoma. Oncogene. 2011; 30(12): 1390-401.

23. Movassagh M, Choy MK, Goddard M, Bennett MR, Down TA, Foo RS. Differential DNA methylation correlates with differential expression of angiogenic factors in human heart failure. PLoS One. 2010; 5(1): e8564.

24. Tsui DW, Lam YM, Lee WS, Leung TY, Lau TK, Lau ET, et al. Systematic identification of placental epigenetic signatures for the noninvasive prenatal detection of Edwards syndrome. PLoS One. 2010; 5(11): e15069.

25. Weber M, Davies JJ, Wittig D, Oakeley EJ, Haase M, Lam WL, et al. Chromosome-wide and promoter-specific analyses identify sites of differential DNA methylation in normal and transformed human cells. Nat Genet. 2005; 37(8): 853-62.

26. Novak JP, Kim SY, Xu J, Modlich O, Volsky DJ, Honys D, et al. Generalization of DNA microarray dispersion properties: microarray equivalent of t-distribution. Biol Direct. 2006; 1: 27.

27. Zhang S. An improved nonparametric approach for detecting differentially expressed genes with replicated microarray data. Stat Appl Genet Mol Biol. 2006; 5: Article30.

28. Cheng AS, Culhane AC, Chan MW, Venkataramu CR, Ehrich M, Nasir A, et al. Epithelial progeny of estrogen-exposed breast progenitor cells display a cancer-like methylome. Cancer Res. 2008; 68(6): 1786-96.

29. Tomazou EM, Rakyan VK, Lefebvre G, Andrews R, Ellis P, Jackson DK, et al. Generation of a genomic tiling array of the human major histocompatibility complex (MHC) and its application for DNA methylation analysis. BMC Med Genomics. 2008; 1: 19.

30. Liu Y, Balaraman Y, Wang G, Nephew KP, Zhou FC. Alcohol exposure alters DNA methylation profiles in mouse embryos at early neurulation. Epigenetics. 2009; 4(7): 500-11.

31. Murphy DM, Buckley PG, Bryan K, Das S, Alcock L, Foley NH, et al. Global MYCN transcription factor binding analysis in neuroblastoma reveals association with distinct E-box motifs and regions of DNA hypermethylation. PLoS One. 2009; 4(12): e8154.

32. Takeshima H, Yamashita S, Shimazu T, Niwa T, Ushijima T. The presence of RNA polymerase II, active or stalled, predicts epigenetic fate of promoter CpG islands. Genome Res. 2009; 19(11): 1974-82.

33. Feber A, Wilson GA, Zhang L, Presneau N, Idowu B, Down TA, et al. Comparative methylome analysis of benign and malignant peripheral nerve sheath tumors. Genome Res. 21(4): 515-24.

34. Jia J, Pekowska A, Jaeger S, Benoukraf T, Ferrier P, Spicuglia S. Assessing the efficiency and significance of Methylated DNA Immunoprecipitation (MeDIP) assays in using in vitro methylated genomic DNA. BMC Res Notes. 2010; 3: 240.

35. Jin SG, Kadam S, Pfeifer GP. Examination of the specificity of DNA methylation profiling techniques towards 5-methylcytosine and 5-hydroxymethylcytosine. Nucleic Acids Res. 2010; 38(11): e125.

36. Nair SS, Coolen MW, Stirzaker C, Song JZ, Statham AL, Strbenac D, et al. Comparison of methyl-DNA immunoprecipitation (MeDIP) and methyl-CpG binding domain (MBD) protein capture for genome-wide DNA methylation analysis reveal CpG sequence coverage bias. Epigenetics. 2011; 6(1): 34-44.

37. Rajendram R, Ferreira JC, Grafodatskaya D, Choufani S, Chiang T, Pu S, et al. Assessment of methylation level prediction accuracy in methyl-DNA immunoprecipitation and sodium bisulfite based microarray platforms. Epigenetics. 2011; 6(4): 410-5.

38. Robinson MD, Stirzaker C, Statham AL, Coolen MW, Song JZ, Nair SS, et al. Evaluation of affinity-based genome-wide DNA methylation data: effects of CpG density, amplification bias, and copy number variation. Genome Res. 2010; 20(12): 1719-29.

39. Yang L, Zhang K, Dai W, He X, Zhao Q, Wang J, et al. Systematic evaluation of genome-wide methylated DNA enrichment using a CpG island array. BMC Genomics. 2011; 12: 10.

40. Irizarry RA, Ladd-Acosta C, Carvalho B, Wu H, Brandenburg SA, Jeddeloh JA, et al. Comprehensive high-throughput arrays for relative methylation (CHARM). Genome Res. 2008; 18(5): 780-90.

41. Chen LS, Storey JD. Eigen-R2 for dissecting variation in high-dimensional studies. Bioinformatics. 2008; 24(19): 2260-2.
