# Supplementary material for: Association of Childhood Chronic Physical Aggression with a DNA Methylation Signature in Adult Human T Cells
Source: PLoS One. 2014 Apr 1;9(4):e89839. doi: 10.1371/journal.pone.0089839 (PMC3972178; doi:10.1371/journal.pone.0089839)
Supplement: Table S1 — Top list of affected biological functions enriched with genes whose methylation is associated with aggression from Ingenuity Pathway (12) analysis (n = 448 genes). All of the p values were calculated using a right tailed Fisher's exact test and corrected for multiple comparison with the Benjamini-Hochberg method. Significance threshold were p = 0.05. (DOCX) [file pone.0089839.s005.docx]

**Table S1. Top list of affected biological functions enriched with genes whose methylation is associated with aggression from IPA analysis (n=448 genes).** All of the p values were calculated using a right tailed Fisher's exact test and adjusted for multiple comparison to obtain false discovery rates using the Benjamini-Hochberg algorithm.

| **Rank** | **Category** | **Functions Annotation** | **FDR** | **Regulation z-score** |
| --- | --- | --- | --- | --- |
| **Enriched with genes less methylated in CPA** | | |  |  |
| **1** | Psychological Disorders | hyperactive behavior | 0.001 | -2.3 |
| **2** | Cell-To-Cell Signaling and Interaction | activation of cells | 0.02 | -1.9 |
| **3** | Metabolic Disease | adiposity | 0.008 | -1.8 |
| **4** | Inflammatory Response | chemotaxis of phagocytes | 0.03 | -1.7 |
| **5** | Cell Death | apoptosis of hepatoma cell lines | 0.03 | -1.5 |
| **6** | Organismal Development | mass of organism | 0.04 | -1.4 |
| **7** | Organismal Injury and Abnormalities | bleeding | 0.03 | -1.3 |
| **8** | Cardiovascular Disease | dilated cardiomyopathy | 0.03 | -1.0 |
| **9** | Cellular Assembly and Organization | quantity of plasma membrane projections | 0.03 | -0.8 |
| **10** | Cellular Compromise | damage of neurons | 0.03 | -0.8 |
| **11** | Carbohydrate Metabolism | synthesis of polyols | 0.02 | -0.7 |
| **12** | Behavior | behavior | 0.02 | -0.6 |
| **13** | Cell Morphology | polarization of leukocytes | 0.002 | -0.5 |
| **14** | Nutritional Disease | weight gain | 0.006 | -0.5 |
| **15** | Cell Morphology | polarization of cells | 0.007 | -0.5 |
| **Enriched with genes more methylated in CPA** | | |  |  |
| **1** | Neurological Disease | encephalopathy | 0.0008 | 2.0 |
| **2** | Cellular Growth and Proliferation | proliferation of cancer cells | 0.03 | 1.8 |
| **3** | Cellular Growth and Proliferation | proliferation of blood cells | 0.03 | 1.4 |
| **4** | Cell-mediated Immune Response | differentiation of helper T lymphocytes | 0.03 | 1.3 |
| **5** | Cellular Movement | invasion of lung cancer cell lines | 0.005 | 1.3 |
| **6** | Nutritional Disease | obesity | 0.006 | 1.2 |
| **7** | Hematological Disease | neutrophilia | 0.006 | 1.0 |
| **8** | Gastrointestinal Disease | disorder of pancreas | 0.03 | 1.0 |
| **9** | Gene Expression | transcription of DNA | 0.01 | 0.9 |
| **10** | Connective Tissue Development and Function | quantity of osteoclasts | 0.02 | 0.8 |
