# Supplementary material for: Association of Childhood Chronic Physical Aggression with a DNA Methylation Signature in Adult Human T Cells
Source: PLoS One. 2014 Apr 1;9(4):e89839. doi: 10.1371/journal.pone.0089839 (PMC3972178; doi:10.1371/journal.pone.0089839)
Supplement: Table S2 — Top list of canonical pathways enriched with genes whose methylation is associated with aggression from IPA analysis (n = 448 genes). All of the p values were calculated using a right tailed Fisher's exact test and corrected for multiple comparison with the Benjamini-Hochberg method. Significance threshold were p = 0.05. (DOCX) [file pone.0089839.s006.docx]

**Table S2. Top list of canonical pathways enriched with genes whose methylation is associated with aggression from IPA analysis (n=448 genes).**

| **Rank** | **Ingenuity Canonical Pathways** | **FDR** | **Gene promoters more or** less **methylated in CPA** |
| --- | --- | --- | --- |
| **1** | PPAR Signaling | 7E-05 | IL33, PPARG, IL1R2, SRA1, C20orf191, IL1RN, **IL1R1**, NRIP1, **PPARGC1A** |
| **2** | Cytokines signaling between Immune Cells | 0.003 | IL33, IL20, IL1RN, IL17F, IL24 |
| **3** | Leukocyte Extravasation Signaling | 0.004 | **GNAI3**, **CTNNA2**, MMP14, **VAV3**, CLDN18, MAPK8, **PLCG1**, **PIK3CB**, CLDN14, **CLDN7** |
| **4** | PPARα/RXRα Activation | 0.006 | PLCZ1, IL1R2, PLCB4, ACVR1, MAPK8, **CLOCK**, **PLCG1**, **IL1R1**, **PPARGC1A** |
| **5** | LXR/RXR Activation | 0.007 | IL33, IL1R2, C20orf191, IL1RN, **SAA4**, ITIH4, **IL1R1** |
| **6** | G-Protein Coupled Receptor Signaling | 0.009 | GPR115, ADORA3, FYN, TRHR, **GPR84**, GPR45, HTR1D, GPR176, AVPR1A, PDE4D, GRM5, PLCB4, **DRD1**, GLP2R, **PIK3CB**, **RXFP3**, FPR3, AGTR1 |
| **7** | FXR/RXR Activation | 0.009 | IL33, PPARG, IL1RN, FETUB, MAPK8, **PPARGC1A** |
| **8** | p70S6K Signaling | 0.010 | PLCZ1, **GNAI3**, PLCB4, **PLCG1**, **PIK3CB**, PLD1, AGTR1 |
| **9** | IL-10 Signaling | 0.011 | IL33, IL1R2, IL1RN, MAPK8, **IL1R1** |
| **10** | Role of Macrophages, Fibroblasts and Endothelial Cells in Rheumatoid Arthritis | 0.014 | PLCZ1, IL33, IL1R2, TLR1, PLCB4, SELE, IL1RN, **PLCG1**, **PIK3CB**, **TCF7L1**, **IL1R1**, **LRP1** |
| **11** | p38 MAPK Signaling | 0.014 | IL33, IL1R2, IL1RN, **IL1R1**, **MKNK2**, HIST2H3D |
| **12** | PI3K Signaling in B Lymphocytes | 0.016 | PLCZ1, FYN, PLCB4, **VAV3**, **PLCG1**, ATF6, **PIK3CB** |
