# Supplementary material for: Association of Childhood Chronic Physical Aggression with a DNA Methylation Signature in Adult Human T Cells
Source: PLoS One. 2014 Apr 1;9(4):e89839. doi: 10.1371/journal.pone.0089839 (PMC3972178; doi:10.1371/journal.pone.0089839)
Supplement: Table S3 — List of transcription regulators showing a significant overlap with genes whose methylation is associated with aggression from IPA analysis (n = 448 genes). Transcription regulators differentially methylated between chronic and normal aggression are shown in bold. Significance threshold were p = 0.05. (DOCX) [file pone.0089839.s007.docx]

**Table S3. List of transcription regulators showing a significant overlap with genes whose methylation is associated with aggression from IPA analysis (n=448 genes).** Transcription regulators differentially methylated between chronic and normal aggression are shown in bold.

| **Transcription Regulator** | **P value of overlap** | **Target gene promoters more or** less **methylated in CPA** |
| --- | --- | --- |
| STAT6 | 1.48E-04 | HIPK2,IL17F,IL1RN,IL24,**IRF4**,**ISG20**,LIFR,MMP14,**NEDD9**,PLD1,SELE,TFEC |
| SWI-SNF | 2.01E-03 | PPARG,SELE |
| FOXH1 | 2.01E-03 | ALDH1A2,**ALDH1A3** |
| **FOXC2** | 4.29E-03 | **ADD1**,**DLL1**,PPARG,**PPARGC1A** |
| SOX3 | 4.90E-03 | **DLL1**,**ZBTB16** |
| NRF1 | 8.13E-03 | **COX5B**,**COX6A1**,IL1RN,**VDAC1** |
| ESR2 | 9.86E-03 | IL20,LTBP1,MMP14,**NEDD9**,SELE,**VAV3** |
| NFATC2 | 1.02E-02 | **IRF4**,PLD1,PPARG,**DSCR1**,SELE |
| HOXA9 | 1.05E-02 | **ARL3**,**CLCN7**,CREM,HBG1,**JUNB**,MBNL1,NEB,SELE |
| PGR | 1.26E-02 | AKR1C3,**ALDH1A3**,CALD1,**IL1R1**,**NEDD9**,PTP4A1,UCK2 |
| FOS | 1.47E-02 | AGTR1,AKR1C3,**ALDH1A3**,**ARID3A**,**DEDD**,FETUB,**HRK**,**JUNB**,LTBP1,MGST1,NBPF11,NRIP1,**PIK3CB**,PLD1,SYT1,**VAV3** |
| SPI1 | 1.57E-02 | **CIITA**,IL1R2,IL1RN,IL24,**IRF4**,SP6,TFEC |
| NKX2-8 | 1.86E-02 | AFP |
| KCNIP3 | 2.00E-02 | CREM,**HRK** |
| CREB1 | 2.44E-02 | **CIITA**,CREM,**ETV3**,**JUNB**,**MKNK2**,PPARG,**PPARGC1A**,RP1,TPO |
| MEOX2 | 2.88E-02 | **LRP1**,MMP14,SELE |
| **RCAN1** | 3.07E-02 | **DSCR1**,SELE |
| MYC | 3.36E-02 | **ADD1**,AFP,**COX5B**,**COX6A1**,**COX7A2L**,**RPL27** |
| RORA | 3.45E-02 | ACOT2,B3GALT1,IL17F,NAT2,NAT8,**PPARGC1A**,SLC41A2 |
| HTT | 3.67E-02 | ACOT2,**ATP2B2**,**CLOCK**,**COX6A1**,DCN,**DLL1**,**DRD1**,**JUNB**,KCNJ1,MGP,MMP14,PDX1,**PITPNM1**,PPARG,**PPARGC1A**,SP6,**STRN4**,**TIAM1**,**VGF** |
| TFIIH | 3.68E-02 | PPARG |
| CREB5 | 3.68E-02 | PPARG |
| TBX6 | 3.68E-02 | **DLL1** |
| BRD8 | 3.68E-02 | PPARG |
| GLIS2 | 3.88E-02 | MGP,MMP14 |
| TP53 | 4.22E-02 | AFP,AGTR1,**DICER1**,FYN,**JUNB**,LTBP1,NLRC4,PPARG,PTP4A1,SPATA18,**UIMC1** |
| RORC | 4.26E-02 | ACOT2,B3GALT1,IL17F,NAT2,NAT8,SLC41A2 |
| LEF1 | 4.27E-02 | KCNIP4,NRCAM,**POU3F2** |
| ZNF217 | 4.29E-02 | **DPP6**,NMNAT2,**DSCR1**,**ST6GAL1** |
| STAT3 | 4.39E-02 | **CIITA**,**DLL1**,HBG1,IL17F,IL1RN,**IRF4**,**ISG20**,ITGB6,**JUNB**,**PPARGC1A**,**SLC9A3** |
| HNF1A | 4.75E-02 | A1CF,AFP,AKR1C3,**ALS2**,ANKS4B,AQP9,FAM107B,ITIH4,MIA2,NDUFS2,PDX1,**SFXN2**,UGT2B11/UGT2B28 |
| MEF2 | 4.77E-02 | IL1RN,**PPARGC1A** |
| MED30 | 4.77E-02 | NDUFS2,**PPARGC1A** |
| **CLOCK** | 4.92E-02 | AVPR1A,**CLOCK**,**JUNB**,MAPK8,MGP,**MKNK2** |
