# Supplementary material for: Association of Childhood Chronic Physical Aggression with a DNA Methylation Signature in Adult Human T Cells
Source: PLoS One. 2014 Apr 1;9(4):e89839. doi: 10.1371/journal.pone.0089839 (PMC3972178; doi:10.1371/journal.pone.0089839)
Supplement: Table S4 — Primers and melting temperature (Tm) used for Q-MeDIP and pyrosequencing. (DOCX) [file pone.0089839.s008.docx]

**Table S4. Primers and melting temperature (Tm) used for Q-MeDIP and pyrosequencing.**

| **Name** | **sequence 5'-3'** | **TM** | **Length** |
| --- | --- | --- | --- |
| **QMeDIP** | | | |
| ZNF336_qMeDIP.Fow | GGCCAAGCTCTCCCTTGCTC | 60 | 122 |
| ZNF336_qMeDIP.Rev | CGGTGTTCCAGACAGGCAGG |  |  |
| FYN_qMeDIP.Fow | TGGACCGTCTGTAGGCAGGTGT | 60 | 188 |
| FYN_qMeDIP.Rev | GGCAGTGGCTGGGGAGATGG |  |  |
| TMCO7_qMeDIP.Fow | TCCAGGGTGTGCTGGTGGAGA | 60 | 176 |
| TMCO7_qMeDIP.Rev | ACTCCTCTGCGTTTCACACAAGTT |  |  |
| ANKRD50_qMeDIP.Fow | TGCCAAAGGTGAAGTACGTGGA | 60 | 171 |
| ANKRD50_qMeDIP.Rev | TCTTGGTTTTAAGACGTCCGGGT |  |  |
| ZNF808_qMeDIP.Fow | ACGTTCAGTCAGAAGGCAACCCT | 60 | 180 |
| ZNF808_qMeDIP.Rev | ACGCACGAAAGCCTTGTCACA |  |  |
| TGIF1_qMeDIP.Fow | TTGCTTGGCCTTGAGGCAGC | 60 | 120 |
| TGIF1_qMeDIP.Rev | CGTTGCTTGGTGCTGGCGGA |  |  |
| ACOT2_qMeDIP.Fow | CTATTAGCCGGGCGCGGTGG | 60 | 187 |
| ACOT2_qMeDIP.Rev | CCCACATCGAAGGGTGCTGGC |  |  |
| ITGB6_qMeDIP.Fow | TCACGTACAAGGTAAGACCTGCGT | 60 | 184 |
| ITGB6_qMeDIP.Rev | TCACTGTGGGAACTGCCCGGT |  |  |
| RNF219_qMeDIP.Fow | TCACCCCATCCAATCCCCGAA | 60 | 163 |
| RNF219_qMeDIP.Rev | GGGAGCTGCAAGATTCCTGCGA |  |  |
| GRM5_qMeDIP.Fow | TGGTACATGGGGACAAAATGGTCCT | 60 | 131 |
| GRM5_qMeDIP.Rev | GCATGGTGGGGAAAATTCAGGAGGG |  |  |
| OR1J4_qMeDIP.Fow | ACATGAAGGCTCATGACTAGCCTGA | 60 | 171 |
| OR1J4_qMeDIP.Rev | TCGCCAAGAGAGTAGAGGGTTTGA |  |  |
| AVPR1A_qMeDIP.Fow | ATGGCATCCAGCACAACCAG | 60 | 237 |
| AVPR1A_qMeDIP.Rev | TGTTGGTGGATACAGTTGGAAGA |  |  |
| IL1R2_qMeDIP.Fow | TGGGGCCACAGTTGGGCAAAA | 60 | 230 |
| IL1R2_qMeDIP.Rev | GGGTGGTGTCACCCGCTCAC |  |  |
| IL1RN_qMeDIP.Fow | GCTTCTCGCAGTGGGGCAGG | 60 | 201 |
| IL1RN_qMeDIP.Rev | CCCAGAGGGTCGGCAGATCGT |  |  |
| IL31_qMeDIP.Fow | GAAGCCTTCGACCCGCCACA | 60 | 214 |
| IL31_qMeDIP.Rev | AGCCCAGGGTTGACAGACACGA |  |  |
| IL33_qMeDIP.Fow | ACTATGCATGATTGTATGTGCT | 60 | 204 |
| IL33_qMeDIP.Rev | GGCTTTGGTCAATGGATTGC |  |  |
| JUNB_qMeDIP.Fow | TGGGTCATGCAGCTCCACCCA | 60 | 268 |
| JUNB_qMeDIP.Rev | CGCAGCAAGTGGGGTGAGGG |  |  |
| OR13C8_qMeDIP.Fow | TCACAGAGTTCTGTTGGAGATCAGT | 60 | 152 |
| OR13C8_qMeDIP.Rev | GCAGGATCATCAGGTACATCCACA |  |  |
| PCDHB8_qMeDIP.Fow | TGCCCAAAACTCAGTTAGCATTGT | 60 | 131 |
| PCDHB8_qMeDIP.Rev | TGCCTGTATGTACCCTGGGACT |  |  |
| SLC41A2_qMeDIP.Fow | TGCGGTTCTGATTCATCTGCTGT | 60 | 247 |
| SLC41A2_qMeDIP.Rev | TGCCTGTCTTCTTGGTTTTTCTGGT |  |  |
| **Pyrosequencing** | | | |
| ITGB6_bis_out.Fow | GGATTGAATTGTTTTGTTTGTTTTT | 55 | 286 |
| ITGB6_bis_out.Rev | TCCTCATTAAAATACCTATACAACCTTTAT |  |  |
| ITGB6_bis_nest.Fow | TTGTTTGTTTTTTTTATTTTTAGGAAG | 60 | 224 |
| ITGB6_bis_nest.Rev | /5Biosg/AAAACATTCACTATAAAAACTACCC |  |  |
| ITGB6_bis_seq1.Fow | AGGAAGGAATGATTA |  |  |
| ITGB6_bis_seq2.Fow | ATTATAGATAAATAT |  |  |
| GRM5_bis_out.Fow | ATTATTTAATTAAATGGTATATGGGGATAA | 55 | 204 |
| GRM5_bis_out.Rev | AAACTAACAAAATCAACAAAAAAACC |  |  |
| GRM5_bis_nest.Fow | TATGGGGATAAAATGGTTTTTTAGA | 60 | 150 |
| GRM5_bis_nest.Rev | /5Biosg/AAAAAATTCAAACCAATAAAAATAACATAA |  |  |
| GRM5_bis_seq1.Fow | TAATTTTTTGATTTG |  |  |
| OR13C8_bis_nest.Fow | GTTGGAGATTAGTATATGTAATATG | 55 | 163 |
| OR13C8_bis_nest.Rev | /5Biosg/CTAAAATAAAAACTCCATTTCC |  |  |
| OR13C8_bis_out.Fow | TTTTGTTGGAGATTAGTATATGT | 60 | 300 |
| OR13C8_bis_out.Rev | ACCTTTTTCTTTACTACCAA |  |  |
| OR13C8_bis_seq1.Fow | TATGGAAAGGATTAA |  |  |
| OR13C8_bis_seq2.Fow | TTAGATAGTTTTTTT |  |  |
| IL31_bis_nest.Fow | ATTTGGAGGGTAATTTTAGT | 55 | 220 |
| IL31_bis_nest.Rev | /5Biosg/AAAAAAAACAAAACCAACTTC |  |  |
| IL31_bis_out.Fow | GTTTATTTGGAGGGTAATTTTAG | 60 | 298 |
| IL31_bis_out.Rev | CCTAATAAAATAAAATAATACC |  |  |
| IL31_bis_seq1.Fow | GTTTTTTTATTTAGT |  |  |
| IL31_bis_seq2.Fow | TGGTTTTGTTATGGG |  |  |
